# Open science, COVID-19, and the news: Exploring controversies in the circulation of early SARS-CoV-2 genomic epidemiology research

**DOI:** 10.1080/17441692.2021.1896766

**Published:** 2021-03-04

**Authors:** Stephen Molldrem, Mustafa I. Hussain, Anthony K J Smith

**Affiliations:** a Department of Anthropology, University of California, Irvine, CA, USA; b Department of Informatics, University of California, Irvine, CA, USA; c Centre for Social Research in Health, UNSW Sydney, Sydney, Australia

**Keywords:** Molecular epidemiology, pathogen genomics, science and technology studies, media studies, science communication

## Abstract

Some early English language news coverage of COVID-19 epidemiology focused on studies that examined how SARS-CoV-2 (the coronavirus that causes COVID-19) was evolving at the genetic level. The use of phylogenetic methods to analyse pathogen genetic sequence data to understand disease dynamics is called ‘molecular’ or ‘genomic’ epidemiology. Many research groups in this subfield utilise open science practices, which can involve the circulation of early unreviewed findings on publicly-accessible venues online. From March to May 2020, media outlets covered early SARS-CoV-2 genomic studies that claimed to have discovered types of SARS-CoV-2 that had mutated to be more transmissible. We use methods from Science and Technology Studies (STS) to examine three cumulative cases in which unripe facts about SARS-CoV-2 genomics moved out of scientific publics and into mainstream news. The three cases are: (1) ‘A More “Aggressive” Strain of SARS-CoV-2?’, (2) ‘Eight SARS-CoV-2 Strains?’, and (3) ‘A “More Contagious,” “Mutant” Strain?’ In each case, findings were called into question and reporters’ framing was overly sensational. We interpret the COVID-19 pandemic as a ‘stress-test’ for open science practices, and argue that it is important for stakeholders to understand changes in scientific publication and dissemination processes in the wake of the pandemic.

## Introduction

Some early English-language news coverage about COVID-19 focused on how SARS-CoV-2, the coronavirus that causes COVID-19, had evolved at the genetic level (Blanchard, 2020; Vartabedian, 2020; Weise, 2020). The use of phylogenetic methods to analyse mutations in pathogen genetic sequences to understand disease dynamics is called ‘molecular’ or ‘genomic’ epidemiology (Armstrong et al., 2019). In this paper, we use methods from Science and Technology Studies (STS; see, Collins, 1985, 2014; Epstein, 1996; Mirowski, 2018) and new media studies (Ananny & Finn, 2020) to analyse three controversies surrounding early SARS-CoV-2 genomic epidemiology studies.

Prior to and after the pandemic was declared by the World Health Organization on 11 March 2020 (Adhanom, 2020), researchers distributed early and often un-reviewed results of SARS-CoV-2 genomic studies via public websites, preprint repositories, and journals that implemented accelerated COVID-19 review processes. We discuss how media coverage of these papers in a chaotic information environment led to distortions of knowledge. We begin by describing the background for our cases, situating them within the early COVID-19 pandemic and the longer history of open science. We use the cases to explore under-studied processes in the practice of genomic epidemiology, scientific publication, and open science.

### SARS-CoV-2, genomic epidemiology, and the news

Genomic epidemiology is a subfield with applications in infectious disease research, treatment, and control (Armstrong et al., 2019; Molldrem & Smith, 2020). While interest in pathogen genomics is usually limited to scientific publics, studies are sometimes covered in the mainstream press – often in stories about the emergence of a purportedly new, more virulent, or treatment-resistant strain of a pathogen (Davis et al., 2018). This is often the case whether or not this framing reflects the underlying science (Blick et al., 2007; Thaczuk, 2007).

Pathogen transmission stories are sometimes sensationalised by scientists, public health authorities, and reporters (McKay, 2017; Watney, 1996). Examples of media sensationalism have arisen in response to Swine Flu (Davis & Lohm, 2020), antiviral-resistant HIV (Blick et al., 2007), and antibiotic-resistant sexually transmissible infections (Davis et al., 2018). The examples of news coverage of SARS-CoV-2 genomic studies that we discuss reflect these underlying dynamics, which structure media coverage of infectious diseases, as well as changes in the sociotechnical makeup of news organisations (Ananny & Finn, 2020; Hastings, 2020) and open science infrastructures (Leonelli et al., 2015; Mendez et al., 2020; Mirowski, 2018).

Because each pathogen has distinct patterns of evolution, each also presents its own challenges when communicating with the public. In the case of SARS-CoV-2, scientific and media narratives about ‘mutations,’ ‘types,’ ‘strains,’ and ‘variants’ have ranged from attempts to establish the geographical origin of the virus to accounts of the emergence of purportedly more transmissible types (Blanchard, 2020; Braine, 2020; Korber et al., 2020a; Tang et al., 2020; Vartabedian, 2020; Weise, 2020; World Health Organization, 2020). The availability of SARS-CoV-2 genetic sequence data in open datasets, the existence of established pathogen phylogenetic research groups, along with methods deployed as open source software tools, led to an early blossoming of genomic discourse about SARS-CoV-2, along with controversies.

### Open science practice and preprint repositories

Open science is a set of norms and best practices that emerged in the 1990s and became dominant in some fields during the 2010s (Leonelli et al., 2015; Mendez et al., 2020; Mirowski, 2018). Open science generally favours researchers making their findings, data, and methods publicly accessible for scrutiny and re-use, along with other practices designed to support reproducibility and speedy dissemination (Leonelli et al., 2015; Mirowski, 2018; Willinsky, 2005). The practice of posting unreviewed scientific papers (called ‘preprints’) on public online repositories prior to peer review is one component of open science (Collins, 2014; Fry et al., 2019; Mendez et al., 2020; Mirowski, 2018; Sheldon, 2018).

Posting preprints has been promoted as a way to sidestep long waiting times for peer review, so that useful results can be cited quickly (Fry et al., 2019). Many genomic epidemiology research groups post their manuscripts on preprint servers such as arXiv (pronounced ‘archive’), medRxiv, and bioRxiv prior to peer review (e.g. Korber et al., 2020a). Some also post results on other digital platforms used by researchers (e.g. MacLean et al., 2020a), or on personal blogs and websites (e.g. Bedford, 2020). Competing to share research findings in a timely manner, some journals implemented accelerated peer review processes, both prior to and during the COVID-19 pandemic (Callaway, 2020; Coudert, 2020; De Floriani, 2020). These structures and practices have contributed to an increasingly complex scientific information ecosystem.

There are important questions to be addressed regarding how the process of scientific publication has been changing due to preprint practices, accelerated review, and open science platforms (Hadfield et al., 2018; Mirowski, 2018). For example, if a preprint can be downloaded by anyone with internet access, is it not public, and therefore already published (Malički & Marušić, 2014)? Increased permeability between scholarly circles, the news media, and the lay public have profoundly altered information and knowledge economies.

Sheldon (2018) suggested that preprints would lead to the findings of unreviewed-yet-news-worthy papers to be prematurely and widely circulated by news media. Indeed, preprint servers have facilitated the spread of unverified or false information. Since its inception in 1989, the administrators of arXiv – a repository popular among high-energy physicists – have developed a complex moderation system combining algorithmic filtration with manual moderation to relegate fringe topics such as cold fusion, quantum consciousness, and paranormality to the ‘general physics’ category, where scientists familiar with the categorisation system may ignore such work while allowing it to remain online, holding to the ‘open’ spirit of open science (Collins, 2014; Leonelli et al., 2015; Reyes-Galindo, 2016). Consequently, in 2013, supposed evidence for cold fusion – which could revolutionise nuclear power if realised – circulated via arXiv and was reported by a number of news outlets (arXiv Trackbacks, 2013; Collins, 2014; Diep, 2013; Hambling, 2013). The news was rightly reported with scepticism; the cold fusion controversy had been settled several times prior, and did not bear out in 2013.

The problem of premature circulation is not exclusive to preprints. For example, a peer-reviewed article published in *Science* claimed that a microbe used arsenic in its DNA (Wolfe-Simon et al., 2011), and the *BBC* cited the article as potential evidence for extraterrestrial life (BBC Staff, 2010). The scientific paper later met serious challenges in journal correspondence and the scientific press (BBC Staff, 2012).

Many scientists responded to the COVID-19 pandemic with a sense of urgency, prioritising the sharing of timely information. As a result, problems related to preprint practices and processes such as accelerated review became acutely visible. For example, the site Publons encouraged users to provide quantitative scores of preprints to assist in determining which were trustworthy (Publons, 2020). Further, results extracted from preprints were used in several peer-reviewed COVID-19 modelling studies, one of which was published in *The Lancet* (Majumder & Mandl, 2020; Wu et al., 2020). In a third example, mainstream media outlets circulated an un-reviewed claim by a group at Stanford University that the COVID-19 mortality rate was lower than previously estimated (Krieger, 2020), and the findings drew serious challenges (Vogel, 2020). Recent changes in publication norms may have lasting consequences – the following cases offer some clues as to what may be in store.

## Materials and methods

To investigate how early genomic studies about SARS-CoV-2 circulated among scientists and in the mainstream press, we undertook a series of scientific controversy studies, wherein the major lines of debate and actors in a disagreement are considered alongside each other to illuminate underlying forces that shape scientific knowledge (e.g. Collins, 1985; Epstein, 1996; Molldrem & Smith, 2020). As scholars working across STS, critical bioethics, health sociology, and health informatics – and who collaborate with genomic epidemiologists – we possess ‘interactional expertise’ in disease phylogenetics and open science (Collins, 2014). Such expertise is the basis for much scholarship in the sociology of scientific knowledge, including our analysis. Following our cases, we discuss broader implications.

The cases were selected and constructed by the first author through sustained attention to early SARS-CoV-2 genomic epidemiology papers and associated news stories early in the pandemic, primarily from February to May 2020. He undertook this as part of his ongoing research on pathogen genomics. By attending closely to early scientific papers and news coverage of SARS-CoV-2 genomics, the first author identified key story themes and papers that generated news coverage, followed the stories closely, and catalogued them as they developed. He also immersed himself in publicly-accessible online fora where genomic epidemiologists were discussing or publishing about SARS-CoV-2 genomics. These sites included Virological.org, NextStrain.org, several investigators’ websites and blogs, select GitHub repositories, Twitter, preprint websites, and the COVID-19 Open Research Dataset community. The cases were also selected based on volume of citations (based on Google Scholar) and media coverage of the studies in question as events unfolded (based on Google News). The second author used the Internet Archive (www.archive.org) to retrieve additional information about dates and times.

Each case involves three elements related to the communication of genomic epidemiology results to lay publics in a pandemic context. First, each episode involved an accelerated public release of early results of genomic SARS-CoV-2 research, including via a fast-tracked peer review process, open science platform, or preprint repository. Second, in each case, news media covered the original studies. Third, the science was challenged by other scientists or undermined by how reporters framed the findings. Taken together, the three cases highlight central issues in the circulation of early SARS-CoV-2 genomic research, particularly around contested claims that SARS-CoV-2 had evolved to be more transmissible.

Knowledge about the evolution of SARS-CoV-2 continued to develop after May 2020, when our case studies end. Of the cases we consider, only claims made by the paper in the third case bore out consequentially on the question of greater transmissibility. Specifically, the preprint considered in case three (Korber et al., 2020a), described a mutation (D614G) that was eventually recognised by WHO as a more transmissible variant (World Health Organization, 2020). The purpose of our paper is not to describe the evolution of SARS-CoV-2, but to illuminate how controversies about the evolution of SARS-CoV-2 shaped early knowledge and relevant information economies.

## Findings

### Case 1: a more ‘aggressive’ strain of SARS-CoV-2?

On 3 March 2020, Tang et al. (2020), a group of scientists based at several Chinese institutions, published ‘On the origin and continuing evolution of SARS-CoV-2’ in the *National Science Review*, published by Oxford University Press (National Science Review, n.d.). The paper was one of the first genomic epidemiology studies published after the onset of the first COVID-19 emergency in Wuhan, China – the city that current scientific consensus cites as the location where SARS-CoV-2 first ‘spilled over’ from bats to humans in late 2019 (Andersen et al., 2020). The authors used phylogenetic methods to analyse 103 SARS-CoV-2 sequences to examine disease dynamics and transmission patterns. The sequences came from around the world, with a plurality from Wuhan.

Tang et al. (2020) retrieved the sequence data from the Global Initiative on Sharing All Influenza Data (GISAID), one entity coordinating the sharing of SARS-CoV-2 pathogen genomic data. The manuscript was received, reviewed, and published in four days; the cover page of the original un-typeset published version stated: ‘Received: 25-Feb-2020; Revised: 28-Feb-2020; Accepted: 29-Feb-2020’ (Tang et al., 2020).^1^ Given the short period when compared to normal peer review, we infer this was an accelerated review process.

A central claim of Tang et al. (2020) was that there were two dominant ‘types’ of the novel coronavirus: ‘S’ and ‘L.’ The authors characterised ‘L’ as more ‘aggressive,’ stating that it had ‘potentially higher transmission and/or replication rates.’ In addition to working from a small number of sequences from many jurisdictions, the authors used questionable methodologies to make assertions about the evolution of ‘L’ from ‘S’ as well as transmission directionality within their sample. This involved re-identifying two cases – though not by name. The authors used demographic data from the GISAID entries and cited a January 2020 press release from the U.S. Centers for Disease Control and Prevention (CDC) and a news report from Australia.^2^ The authors described how they cross-referenced information in those documents with the sequences to make inferences about the travel history of particular entries. They then extrapolated from this to make claims about patterns of global SARS-CoV-2 viral mutation (Tang et al., 2020).

**Figure 1.**
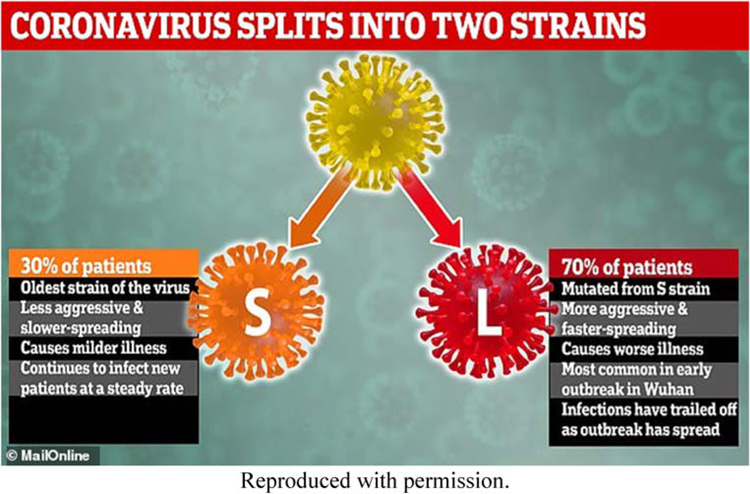
*Daily Mail* Infographic about Tang et al. (2020)’s ‘two types’ finding.

Tang et al.’s (2020) findings were picked up widely by news media. One notable example is a 5 March 2020 article in *The Daily Mail*, a British tabloid. The headline blared: ‘TWO strains of the killer coronavirus are spreading around the world – and 70% of infected patients have caught the more aggressive and contagious type, study claims’ (Blanchard, 2020). The story included an infographic that communicated this finding (Figure 1).

On 5 March 2020,*The International Business Times* published an article titled ‘Coronavirus Mutation Confirmed: Scientists Found Two Types Of COVID-19 Infecting World’ (Villasanta, 2020). *CNBC* published a story titled ‘Chinese scientists identify two strains of the coronavirus, indicating it’s already mutated at least once’ (Meredith, 2020). There were dozens of similar headlines. In all three examples, the journalists caution that the paper was from a small study. However, there was overwhelming emphasis on the greater transmissibility of the L-type strain that Tang et al. (2020) claimed. No stories we reviewed included quotes from interviews with the study authors.

Also on 5 March 2020, two days after Tang et al. (2020) was published, researchers based at the University of Glasgow Centre for Virus Research posted a reply on the website Virological.org titled ‘Response to “On the origin and continuing evolution of SARS-CoV-2”’ (MacLean et al., 2020a). Virological.org is an open source platform used by genomic epidemiologists; it describes itself as ‘[a] discussion forum for analysis and interpretation of virus molecular evolution and epidemiology’ (Virological.org, n.d.). Virological.org was an early hub of SARS-CoV-2 genomic research activity.

MacLean et al. (2020a) critiqued Tang et al.’s (2020) sample size and methodology. They first argued that Tang et al. (2020) had not identified two distinct types of SARS-CoV-2, but rather that they had erroneously assigned significance to benign mutations that, while potentially epidemiologically informative, had no bearing on virulence or transmissibility. MacLean et al. (2020a) wrote: ‘One nonsynonymous mutation, which has not been assessed for functional significance, is not sufficient to define a distinct “type” nor “major type.”‘ They further cited the circulation of the ‘more aggressive type’ discourse in the news media as a reason for their response, writing that ‘[e]vidence from the widespread media uptake … and many comments on social media in response to this article, suggests that the unsupported claims made by Tang et al. have already spread undue fear’ (MacLean et al., 2020a).

MacLean et al. (2020a) also took issue was with Tang et al. (2020)’s statistical methodology: ‘The numbers in [Tang et al.’s] figure do not make sense … For two mutations to have derived frequencies greater than 95%, there would need to be a small number of samples which branch as a sister lineage to the rest of the outbreak tree. However, this is not the case.’ MacLean et al. (2020a) also stated that, even if one agreed with Tang et al.’s (2020) analysis, ‘[w]hen interpreting their results, Tang et al. do not consider that sequencing error could be a driver of a relative excess of’ mutations (MacLean et al., 2020a). Sequencing error and laboratory contamination are common problems in genomic epidemiology. MacLean et al. (2020a) concluded:

[t]aken together, Tang’s [*sic*] analysis tells us absolutely nothing about purifying selection within the viral outbreak … Given these flaws, we believe that Tang et al. should retract their paper, as the claims made in it are clearly unfounded and risk spreading dangerous misinformation at a crucial time in the outbreak.

Further debate unfolded on Virological.org, which included other scientists and several coauthors of Tang et al. (2020). When offered to have a published debate in *National Science Review*, MacLean et al. (2020a) declined, writing that ‘As Jian Lu on behalf of Tang et al. has chosen to post their response here, we see no reason to replicate our critique in the [*National Science Review*] journal.’ By 12 March 2020, the un-typeset version of Tang et al. (2020) had been modified with an addendum,^3^ which read in part:

while we have shown that the two lineages naturally co-exist, we provided no evidence supporting any epidemiological conclusion regarding the virulence or pathogenicity of SARS-CoV-2 … corrections will be made in the print version of this paper to avoid being misleading.

As of 5 September 2020, the language about the ‘aggressiveness’ of the ‘L’ type was still in Tang et al. (2020), and Google Scholar indicated 483 citations. However, as of 14 October 2020 the original un-typeset version of the article had been replaced with a heavily revised typeset version with the ‘aggressive’ language removed and other changes.^4^

This controversy is instructive partly because it shows how open science practices have affected the traditional model of peer review, response, emendation, and retraction. Notably, MacLean et al. (2020a) declined to publish a response in the *National Science Review*, while still compelling Tang et al. (2020) to add an addendum, which fell short of MacLean et al. (2020a)’s call for retraction. MacLean et al. (2020a) went on to publish a version of their critique as a ‘Reflection’ in the journal *Virus Evolution* in May 2020 (see, MacLean et al., 2020b); however, the media controversy over Tang et al. (2020) had already mostly played out by this time. From the perspective of public discourse about SARS-CoV-2 genomics, this episode shows some drawbacks of using vehicles other than fully peer-reviewed publication to affect discourse on a matter of intense public attention.

### Case 2: Eight SARS-CoV-2 strains?

In this case, we consider coverage of SARS-CoV-2 and the NextStrain.org project in *USA Today*. Established in 2018, NextStrain.org is housed at the University of Washington in Seattle (Hadfield et al., 2018), and attracted media attention early in the COVID-19 pandemic. The interactive website conceptualises itself as a platform to ‘provide a *real-time* snapshot of evolving pathogen populations and to provide interactive data visualizations to virologists, epidemiologists, public health officials, and community scientists’ (NextStrain.org, n.d., emphasis in the original).

The NextStrain.org team was credited with generating a range of novel insights during the early COVID-19 pandemic, including the early discovery of community transmission in the United States (Bedford, 2020). Investigators associated with NextStrain.org regularly disseminate findings through peer-reviewed papers, but also through non-peer reviewed publications such as blog posts. One such blog post by Trevor Bedford, a NextStrain.org-affiliated investigator, was posted on 2 March 2020, and written in lay terminology (Bedford, 2020). Bedford’s post discussed how, using phylogenetic analysis, his research group was able to identify community transmission, advising members of the public in Seattle to take precautions nine days before the WHO made the formal COVID-19 pandemic declaration (see also, Bedford et al., 2020).

On 27 March 2020, *USA Today* published an article on the purported global spread of ‘eight strains of the coronavirus,’ a claim made without clear citation but implicitly substantiated by NextStrain.org (Weise, 2020). The article opened in sensational, macabre terms:

At least eight strains of the coronavirus are making their way around the globe, creating a trail of death and disease that scientists are tracking by their genetic footprints.

While much is unknown, hidden in the virus’s unique microscopic fragments are clues to the origins of its original strain, how it behaves as it mutates and which strains are turning into conflagrations while others are dying out thanks to quarantine measures …

Labs around the world are turning their sequencing machines … to the task of rapidly sequencing the genomes of virus samples … The information is uploaded to a website called NextStrain.org that shows how the virus is migrating and splitting into similar but new subtypes.

Weise (2020) made the decision to open the article in this way, despite the fact that the two genomic epidemiologists they quoted said that known mutations were only epidemiologically informative and that variation was unlikely related to greater transmissibility. We note again that Weise (2020) did not refer to any source or interviewer to support the claim that there were eight strains of SARS-CoV-2. However, the ‘eight strains’ claim spread widely in the media, leading to a generalised ‘eight strains’ discourse (e.g. Braine, 2020; Racaniello, 2020).

This case is notable for several reasons. First, it shows that using platforms such as blogs, tools like NextStrain.org, and other public-facing venues to share early genomic epidemiology results can enable reporters to make unsubstantiated claims. Further, it shows that expert interviews do not prevent sensational or misleading coverage. Consequently, scientists’ efforts to educate the public via open platforms such as NextStrain.org can contribute to, rather than ameliorate, confusion about the meaning of genomic pathogen data. For example, as we demonstrated in case one, and as re-emerges here in case two, there was a great deal of confusion – among scientists, journalists, and the public – of the properties that would constitute a distinct SARS-CoV-2 ‘strain’ or ‘type’ – (see also, Rambaut et al., 2020).

To this point, on 7 May 2020, virologist Vincent Racaniello of Columbia University wrote a response to both the ‘two strains’ and ‘eight strains’ controversies on his blog. He said that he believed peer review would solve the problems contained in the studies that led to what he considered to be erroneous findings circulating in the information ecosystem:

No doubt you have heard reports of different SARS-CoV-2 strains, but I assure you they are likely wrong. Some time ago it was claimed in China that there were ‘L’ and ‘S’ strains with distinct pathogenicity in humans. Wrong. You will also hear that there are eight circulating strains of the virus. Wrong. These are all isolates. None have been shown to have a distinct biological property, no matter what the preprints claim … if the scientific review process does its job, most of them will simply be reports of new genome sequences with no associated biological changes (Racaniello, 2020).

However, as we discussed in case one, Tang et al. (2020) was not a preprint, but a paper that apparently underwent accelerated peer review. Further, these claims not only circulated via preprints, but also jumped from NextStrain.org into the mainstream press.

### Case 3: A ‘more contagious,’ ‘mutant’ strain?

On 30 April 2020, a team of genomic epidemiologists posted a preprint titled ‘Spike mutation pipeline reveals the emergence of a more transmissible form of SARS-CoV-2’ on the bioRxiv repository (Korber et al., 2020a). The research was led by members of a long-running pathogen sequencing centre: the Los Alamos HIV Sequence Database and Analysis group at Los Alamos National Laboratory (LANL HIV Sequence Database, n.d.).

On 5 May, the *Los Angeles Times* ran a story about the preprint (Vartabedian, 2020). Originally, the headline was ‘A mutant coronavirus has emerged, even more contagious than the original, study says’ (Vartabedian, 2020). Within hours, the headline was changed to the less-sensational ‘Scientists say a now-dominant strain of the coronavirus appears more contagious than original’; the following day, it was modified to read ‘Scientists say a now-dominant strain of the coronavirus could be more contagious than original.’^5^ The journalist, Vartabedian (2020), interviewed members of the study team, uninvolved experts, and also excerpted a Facebook post by first author Bette Korber. Vartabedian (2020) quoted Korber’s Facebook post:

we see a mutated form of the virus very rapidly emerging, and over the month of March becoming the dominant pandemic form … When viruses with this mutation enter a population, they rapidly begin to take over the local epidemic, thus they are more transmissible.

Vartabedian (2020) then quoted Korber et al. (2020a), which suggested that the mutation (D614G) could be leading to greater contagiousness by region:

‘D614G is increasing in frequency at an alarming rate, indicating a fitness advantage relative to the original Wuhan strain that enables more rapid spread,’ the study said.

Still unknown is whether this mutant virus could account for regional variations in how hard COVID-19 is hitting different parts of the world (Vartabedian, 2020).

Vartabedian’s (2020) inclusion of the qualifying phrase ‘Still unknown is whether’ before suggesting the possibility of a SARS-CoV-2 evolutionary trajectory toward greater transmissibility by region is deceptive rhetoric. It presents a hypothetical, putting forward the possibility of an alternative outcome without making it sufficiently clear that there was not yet evidence to say that the alternative outcome (in this case, SARS-CoV-2 evolution toward greater transmissibility by region) was actually occurring.

Vartabedian (2020) also interviewed several scientists who were not authors on the study, including one researcher who brought up a claim that there were two strains circulating on either coast of the United States:

In the United States, doctors had begun to independently question whether new strains of the virus could account for the differences in how it has infected, sickened and killed people, said Alan Wu, a UC San Francisco professor who runs the clinical chemistry and toxicology laboratories at San Francisco General Hospital.

Medical experts have speculated in recent weeks that they were seeing at least two strains of the virus in the U.S., one prevalent on the East Coast and another on the West Coast, according to Wu.

A paper advancing this argument had been published by Brufsky (2020) on 20 April 2020 in the *Journal of Medical Virology*, and was cited in the Korber et al. (2020a) preprint.

Per a heading included with the un-typeset version of Brufsky (2020) on 14 May 2020, the commentary ‘has been accepted for publication and undergone full peer review.’ The comment was a secondary analysis of published studies and NextStrain.org visualisations. Brufsky (2020) drew on Tang et al. (2020) – the main article considered in case one – to support his claim that two different dominant forms of SARS-CoV-2 were circulating on either coast of the United States, and to argue that they ‘may vary in virulence.’ Brufsky (2020) did not mention that Tang et al. (2020) had been amended following MacLean et al.’s (2020a) critique.

The third case study is notable for several reasons. Firstly, it confirms anxieties within the scientific community that preprints will often be covered by journalists in much the same fashion as papers that have been fully reviewed (Malički & Marušić, 2014; Sheldon, 2018). We have shown this to be the case even if reporters note that papers have not been reviewed, as Vartabedian (2020) did regarding Korber et al. (2020a). Secondly, this third case study shows that scientific papers that have been critiqued and even amended – in the case of Tang et al. (2020) – can continue to influence science (e.g. Korber et al., 2020a’s citation of Brufsky, 2020; which relies on Tang et al., 2020). Finally, it shows how the framing of a reporter’s coverage of topics such as whether pathogen mutations are leading to greater transmissibility can sensationalise and distort preliminary findings, particularly when there is uncertainty, disagreement, and confusion among experts.

In April and May 2020, virtually all claims about SARS-CoV-2 toward greater transmissibility were contested (see, Racaniello, 2020). However, as we outlined in ‘Materials and Methods,’ the claim of Korber et al. (2020a) about greater transmissibility owing to the D614G mutation eventually bore out (World Health Organization, 2020). We further note that, in the peer-reviewed version of Korber et al. (2020a) that was eventually published in *Cell* in July 2020, the reference to Brufsky (2020) was removed (see, Korber et al., 2020b). This removal of Brufsky (2020) from the final version (Korber et al., 2020b) does not change the fact that the preprint (Korber et al., 2020a) did cite Brufsky (2020) and generated substantial media coverage and scientific attention, thus affecting the pandemic information and knowledge economies. This aspect of case three in fact reinforces the notion that the release of early results prior to full review can be inadvisable.

Periods of uncertainty followed by verification and consensus about key aspects of pathogens is a central feature of the history of infectious disease science (Epstein, 1996). However, the timeframe for these developments has historically occurred on the order of months or years, not weeks or days. Our cases show that open science practices can accelerate the pace of discovery and dissemination in ways that are both unhelpful and illuminating.

## Discussion

Our results suggest that genomic epidemiologists and other scientists should be cautious about sharing very early results in publicly-accessible venues, and that this is particularly true when publishing about contested topics of significant public interest. Further, our analysis suggests that the COVID-19 pandemic precipitated changes in the practice of genomic epidemiology and open science (Callaway, 2020).

The accelerated pace of dissemination and disagreements about early findings during the pandemic have revealed problematic aspects of open science practice. For example, the ability to unilaterally disseminate early findings provides more opportunities for the outcomes of substandard methodological practice to propagate despite rapid rebuttal. This has the potential to further exacerbate an extant problem in research: flawed and fraudulent papers continue to be cited approvingly, even following retraction (Piller, 2021; Steen, 2011). Issues in this area have persisted despite attention from watchdog groups (Didier & Guaspare-Cartron, 2018). The continued favourable citation of retracted COVID-19 papers requires further investigation. The first case demonstrates the inappropriate propagation effect most clearly, regarding Tang et al. (2020) and fallout from the paper, the effects of which also appear in the NextStrain.org and ‘mutant virus’ cases.

Our findings have relevance for practitioners, policymakers, and for social scientists and humanists studying transformations in scientific knowledge-production in the wake of COVID-19. It is critical for a wide array of stakeholders to understand how COVID-19 knowledge economies and overall information environments are constituted, and to grasp how shifts in research practices may have mixed consequences for the development of scientific knowledge and literacy in the general public.

Several months after our case studies concluded in May 2020, a variant of SARS-CoV-2 showing strong signs of elevated transmissibility was identified in the UK, leading to further public health measures to contain it (Lauring & Hodcroft, 2021; World Health Organization, 2020). False alarms about pathogen mutations before definitive confirmation may cultivate a ‘cry wolf’ effect in regard to pathogen phylogenetics (Nerlich & Koteyko, 2012), potentially leading public health actors and the public to become less responsive to genomic epidemiologists’ warnings when a new, genuinely more dangerous variant does appear. Whether this has occurred in regard to SARS-CoV-2 warrants further study.

The COVID-19 pandemic may be viewed as a kind of ‘stress-test’ for some open science practices (Roiter, 2010). In our cases, we show the utility of open science practices in disseminating early results quickly in ways that can be helpful, while also revealing some limits and harms. In some cases of SARS-CoV-2 genomic epidemiology, open science practices have benefitted the public health response (see, World Health Organization, 2020). However, the lack of a clear process for dissemination – in combination with a noisy mid-pandemic information environment – arguably compromised the quality of some scientific knowledge on issues of immense importance. This is clearest in the first case, with the rapid publication of Tang et al. (2020) followed immediately by an un-reviewed rebuttal by MacLean et al. (2020a) leading to emendation of Tang et al. (2020). Then, as we showed in cases two and three, problematic claims made by Tang et al. (2020) continued to echo in broader scientific and media discourses, fuelling disputes surrounding the validity of claims about SARS-CoV-2 evolution toward greater transmissibility. COVID-19 has also been taken as an occasion for novel publication practices, including new forms of open peer review (Publons, 2020) and the proliferation of preprints and other early results at unprecedented volumes and velocities (Krieger, 2020; Vogel, 2020).

Reshaping open science in the wake of COVID-19 should mean considering how existing practices have affected public health responses that rely on knowledge generated using these approaches. Open science advocates, organisations that facilitate open science, and governance and funding bodies should accept collective responsibility for sources of harm when they are identified, and work to mitigate them.

We recommend that scientists who utilise open science processes work to recognise the notion that ‘information wants to be free’ has limitations and is, taken by itself, an insufficient theoretical basis for open knowledge practices. At the time of this writing, this notion dominates the various ‘open’ movements (i.e. open source, open science, and open access, see Koch & Jones, 2016; Willinsky, 2005). Scientists who observe open science norms should work to develop a more complex view of professional discretion, and can view the barrier between scientific circles and the public as permeable. If scientists operated on this premise, findings would ideally only be circulated publicly when ripe enough for widespread consumption by non-experts.

Fundamental trade-offs between speed and accuracy are ubiquitous (Sudhir, 2016). Over the arc of our cases, unripe facts that were disseminated through time-saving shortcuts led to confusion in addition to arguably wasted time on subsequent studies; it is not clear that any gain in overall speed was achieved. A slower, steadier pace that prioritises appropriate discretion at each step of the research process may improve accuracy and reduce chaos. Rather than focusing on individual researcher responsibility, we suggest an analysis of structural issues in open science, and for practitioners and institutions to consider calls for ‘slow scholarship’ (Mountz et al., 2015) when assessing how to collectively overcome focuses on rapid output.

New publication and dissemination best practices could be developed. For example, scientists could incorporate public and media relations strategies into their dissemination plans – science communication work that we believe should be supported by funding agencies. Professional societies could establish or improve internal channels for circulating preliminary findings. Additionally, institutional infrastructure for specialised science journalism should be strengthened. These suggestions are starting points; further investigation of potential solutions should be enabled by expanding state funding for social studies of science.

We conclude with a note for STS scholars working on topics related to open science. We have aimed to show that ‘open science’ has changed as an object of analysis and a sign under which knowledge is developed in the wake of COVID-19. Our cases suggest that science studies should focus critical attention on the details of publication and review in open science, and should develop more approaches for doing this work. There is an emergent body of literature analysing open science, with a focus on the political economy of knowledge in open science, data infrastructures (Leonelli et al., 2015; Mirowski, 2018), and professional norms (Willinsky, 2005). As open science practices continue to proliferate and transform, an expanded focus on publication, review, and dissemination processes could shed light on these critical aspects of ‘open’ knowledge-production.

## Notes

1. The original un-typeset PDF is available in the Internet Archive: https://web.archive.org/web/20200305125214/https://academic.oup.com/nsr/advance-article/doi/10.1093/nsr/nwaa036/5775463. Tang et al. (2020) remained un-typeset on the *National Science Review*’*s* website until at least early September 2020.

2. References to the Australian news report were removed from the typeset version of Tang et al. (2020).

3. PDF linked from the following Internet Archive page: https://web.archive.org/web/20200312104954/https://academic.oup.com/nsr/advance-article/doi/10.1093/nsr/nwaa036/5775463.

4. Examining the scope of the emendations to the final version is beyond the scope of this paper.

5. Original headline available on the Internet Archive: https://web.archive.org/web/20200505111912/https://www.latimes.com/california/story/2020-05-05/mutant-coronavirus-has-emerged-more-contagious-than-original. First headline modification: https://web.archive.org/web/20200505155459/https://www.latimes.com/california/story/2020-05-05/mutant-coronavirus-has-emerged-more-contagious-than-original. Second headline modification: https://web.archive.org/web/20200506173840/https://www.latimes.com/california/story/2020-05-05/mutant-coronavirus-has-emerged-more-contagious-than-original.
